# Design Considerations for 3D Printed, Soft, Multimaterial Resistive Sensors for Soft Robotics

**DOI:** 10.3389/frobt.2019.00030

**Published:** 2019-04-30

**Authors:** Benjamin Shih, Caleb Christianson, Kyle Gillespie, Sebastian Lee, Jason Mayeda, Zhaoyuan Huo, Michael T. Tolley

**Affiliations:** ^1^Department of Mechanical and Aerospace Engineering, University of California, San Diego, San Diego, CA, United States; ^2^Department of Nanoengineering, University of California, San Diego, San Diego, CA, United States

**Keywords:** soft sensor, 3D printed, soft skin, resistive sensor, strain sensor, soft robotics

## Abstract

Sensor design for soft robots is a challenging problem because of the wide range of design parameters (e.g., geometry, material, actuation type, etc.) critical to their function. While conventional rigid sensors work effectively for soft robotics in specific situations, sensors that are directly integrated into the bodies of soft robots could help improve both their exteroceptive and interoceptive capabilities. To address this challenge, we designed sensors that can be co-fabricated with soft robot bodies using commercial 3D printers, without additional modification. We describe an approach to the design and fabrication of compliant, resistive soft sensors using a Connex3 Objet350 multimaterial printer and investigated an analytical comparison to sensors of similar geometries. The sensors consist of layers of commercial photopolymers with varying conductivities. We characterized the conductivity of TangoPlus, TangoBlackPlus, VeroClear, and Support705 materials under various conditions and demonstrate applications in which we can take advantage of these embedded sensors.

## 1. Introduction

3D printing has enabled many new design and fabrication approaches for robotics (Lipson and Kurman, 2013). In parallel, a new perspective on the role that materials play in robotic design has altered the building blocks and tools with which we create our robotic systems. The ability to print soft and rigid materials simultaneously using a single machine has expanded the realm of possibilities for fabricating robots, including systems that are biomimetic (Pearson et al., 2011; Kumar et al., 2017; Wang et al., 2017b) and ones that have improved resistance to impact through functional gradients (Bartlett et al., 2015).

In the long term, one goal of 3D printing is the ability to print an entire robot in one go and have it walk itself out of the machine upon completion (Lipson, 2015). Recent work has demonstrated this “get up and walk away" concept. Felton et. al. describe a method for building self-folding machines using laminate structures that fold themselves up in multiple stages and can walk away after receiving electronics and a battery (Felton et al., 2014). Similar in overall concept, MacCurdy et. al. used a multimaterial 3D printer to print fluid-filled bellows directly integrated into the transmission of their locomotive robot, which can also walk immediately after attaching a motor and battery without additional mechanical modifications (MacCurdy et al., 2016). However, neither of these robots possess sensing capabilities for feedback control and learning. In addition, robots that can get up and walk away entirely on their own require a built-in, on-board energy source. The previous robots have batteries added after printing, whereas robots like the EcoBots have a stomach for real digestion that enables them to sustain themselves (Ieropoulos et al., 2010).

Soft robotics is one field that has benefited significantly from 3D printing (Rus and Tolley, 2015). Robot designers can print both the molds for making soft robots (Florez et al., 2014) as well as the soft robots directly (Bartlett et al., 2015). However, a major challenge with soft robots is the development of effective sensors. Soft robots are not constrained to prismatic or revolute motions, and obtaining sensory feedback on these motions requires different types of sensors than those used for rigid robots. In addition, soft robots may require sensors that can be placed on complex surface geometries or embedded within the body.

Much of the soft robot development today has focused on actuation (Conn et al., 2012; Connolly et al., 2017; Miriyev et al., 2017; Kellaris et al., 2018). Sensor design for soft robots is complicated because flexible, compliant robots often have non-planar, complex surfaces that are difficult to cover and sensorize with traditional manufacturing techniques. Proper selection from among the wide range of design parameters (e.g., geometry, material, actuation type, etc.) is critical to the function of soft robots. Conventional rigid sensors can be effective for soft robots with constrained motions (Zhao et al., 2016; Homberg et al., 2018; Scimeca et al., 2018). However, the general case of a soft robot with a high number of degrees of freedom requires capabilities such as out-of-plane twisting. Sensors that are directly integrated into the bodies of soft robots and co-fabricated could help improve both their exteroception and interoception capabilities. We want to move toward highly integrated structural and sensing components as we see in biological human bodies, which may not be possible with discrete, rigid sensors.

Recently, interest in 3D printing soft robots has grown significantly. Previously, research groups have printed various actuators (Drotman et al., 2017; Kalisky et al., 2017) and bodies (Umedachi et al., 2013; Bartlett et al., 2015; MacCurdy et al., 2016) for soft manipulation and locomotion. Alongside the development of soft robot actuators, many groups have become interested in incorporating sensors and closing the control loop for feedback on the robot's interactions with its environment. Felt et. al. designed an inductance-based sensing system to measure and control bellowed continuum joints, by wrapping coils of wires and measuring changes in inductance (Felt et al., 2017). We (Shih et al., 2017) and others (Bilodeau et al., 2015; Farrow and Correll, 2015), have embedded soft sensors for measuring bending into the layers of pneumatic fingers, which enabled the fingers to estimate the shapes of various objects using tactile sensing (Bilodeau et al., 2015; Farrow and Correll, 2015; Zhao et al., 2016; Shih et al., 2017). Homberg et. al. clustered sensor readings from their fingers to identify correspondences to gripper configurations and to classify grasped objects (Homberg et al., 2015), Kim et. al. 3D printed pneumatic pouches and connected them to pressure sensors to sense when the pouches came into contact with external objects (Kim et al., 2015), and Pearson et. al. demonstrated tactile sensing in both compliant, 3D structures and soft, 3D printed actuators (Pearson et al., 2010).

Many groups have also begun experimenting with various fabrication techniques for soft sensors, particularly in the form of skin-like structures (Cheng et al., 2010; Bauer et al., 2014; Sonar and Paik, 2016; Devaraj et al., 2018). Muth et. al. customized a printer for sensor fabrication which embeds conductive material into a partially-cured silicone elastomer substrate that then fully cures and solidifies (Muth et al., 2014). Frutiger et. al. and White et. al have also 3D printed conductive materials for sensors (Frutiger et al., 2015; White et al., 2017). Inspired by mechanoreceptors found within skin, Lipomi et. al. and Tee et. al. developed skin-like sensors that can detect strain and pressure (Lipomi et al., 2011; Tee et al., 2015), and Yin et. al. fabricated a robotic skin that senses shear force (Yin et al., 2017). Multiple groups have explored using deep learning to understand deformations and changes in a soft robot using embedded, soft sensors (Han et al., 2018; Soter et al., 2018; Thuruthel et al., 2019). However, the integration of these skins into real-world robot systems for sensing remains an open problem (Silvera-Tawil et al., 2015). Having a separate skin also requires the robot designer to affix the sensing elements in a separate manufacturing step, limiting the ability to sensorize the complex, dynamic bodies of soft robots.

In this paper, we present:

- A method for printing multiple, soft materials simultaneously to produce a resistive sensing element using a commercial 3D printer, which enables users to incorporate arbitrary sensor geometries into their soft robots.
- Design considerations for creating sensors based on 3D printable conductive materials.
- A demonstration for how we can embed soft, complex-shaped sensors into compliant grippers.

A surprising aspect of what we present is that many researchers are thinking about how to make soft robots with embedded liquid metals, whereas commercially available systems today already allow us to directly print general conductive traces. We hypothesize that current commercial systems already allow for the fabrication of soft actuators with embedded sensors. Not only can existing systems already perform the manufacturing, but this approach also enables designs that would otherwise be very challenging for existing fabrication techniques, e.g., multilayer sensor or complex structures within a 3D body. With traditional lithographic approaches, it is not obvious how to fabricate such designs.

In section 2, we discuss background for the topic. In section 3, we explain the materials and methods that we used for the experiments. In section 4, we present and discuss the results, including the types of sensors that we printed and parameters that contributed to their conductivity, model of the sensitivity to strain, and experimental characterization of the sensors. In section 5, we present potential applications of the 3D printed sensors. Lastly, in section 6, we discuss conclusions and future work.

## 2. Materials and Methods

### 2.1. Design and Fabrication of the Resistive Sensors

Many challenges exist with the integration of sensors into the body of soft robots. Current techniques are often unable to accommodate the elastic, large deformations of soft robots (i.e., the stretching and bending) that arise in applications such as wearable computing and smart textiles (Majidi, 2014).

We can overcome many of these challenges using a commercially available 3D printer (Stratasys Objet350 Connex3) that has the ability to mix conductive and dielectric materials. Many groups have produced actuatable soft robots with this tool: (Umedachi et al., 2013; Bartlett et al., 2015; Kim et al., 2015; MacCurdy et al., 2016; Drotman et al., 2017; Kalisky et al., 2017; Kumar et al., 2017; Wang et al., 2017b; Shih et al., 2018). We investigated whether this printer can also directly embed sensors into soft robot systems.

**Figure 1:**
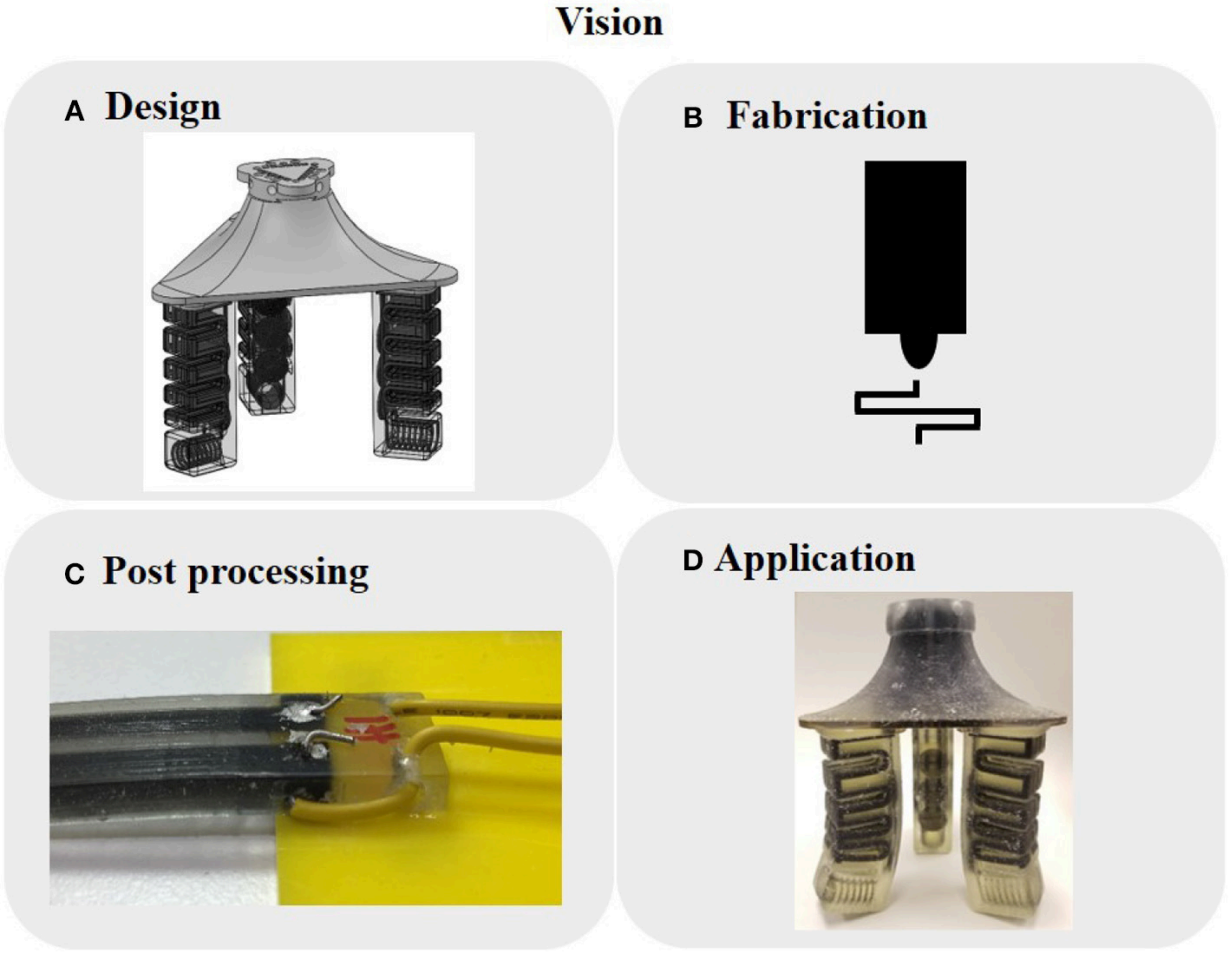
Overall vision of the pipeline for our 3D printed sensors. **(A)** Digitized CAD design with embedded, integrated sensing. **(B)** Multimaterial 3D printer that simultaneously co-fabricates both actuators and sensors. **(C)** Post-processing such as removing support material or wire connections. **(D)** Immediately deployable for real-world applications.

**Figure 2:**
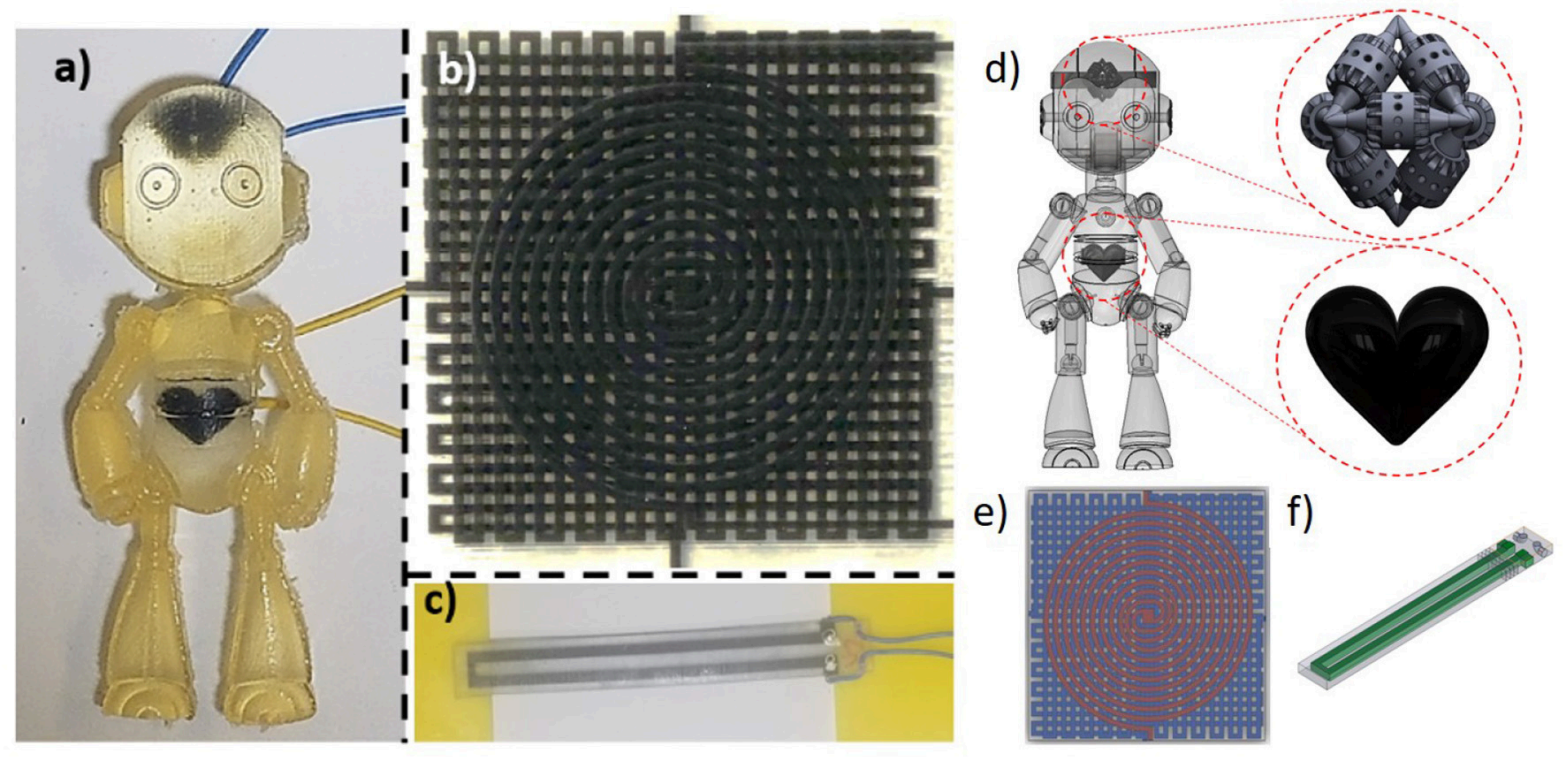
3D printed, resistive, soft sensors and the correspoding CAD drawings. **(a,d)** Humanoid robot-front view. Enlarged drawings of the embedded heart-and brain-shaped sensors. **(b,e)** Multilayer strain and pressure sensor-top view. **(c,f)** Uniaxial strain sensor with mechanical strain relief and functional gradient to improve wire interface-isometric view.

**Figure 3:**
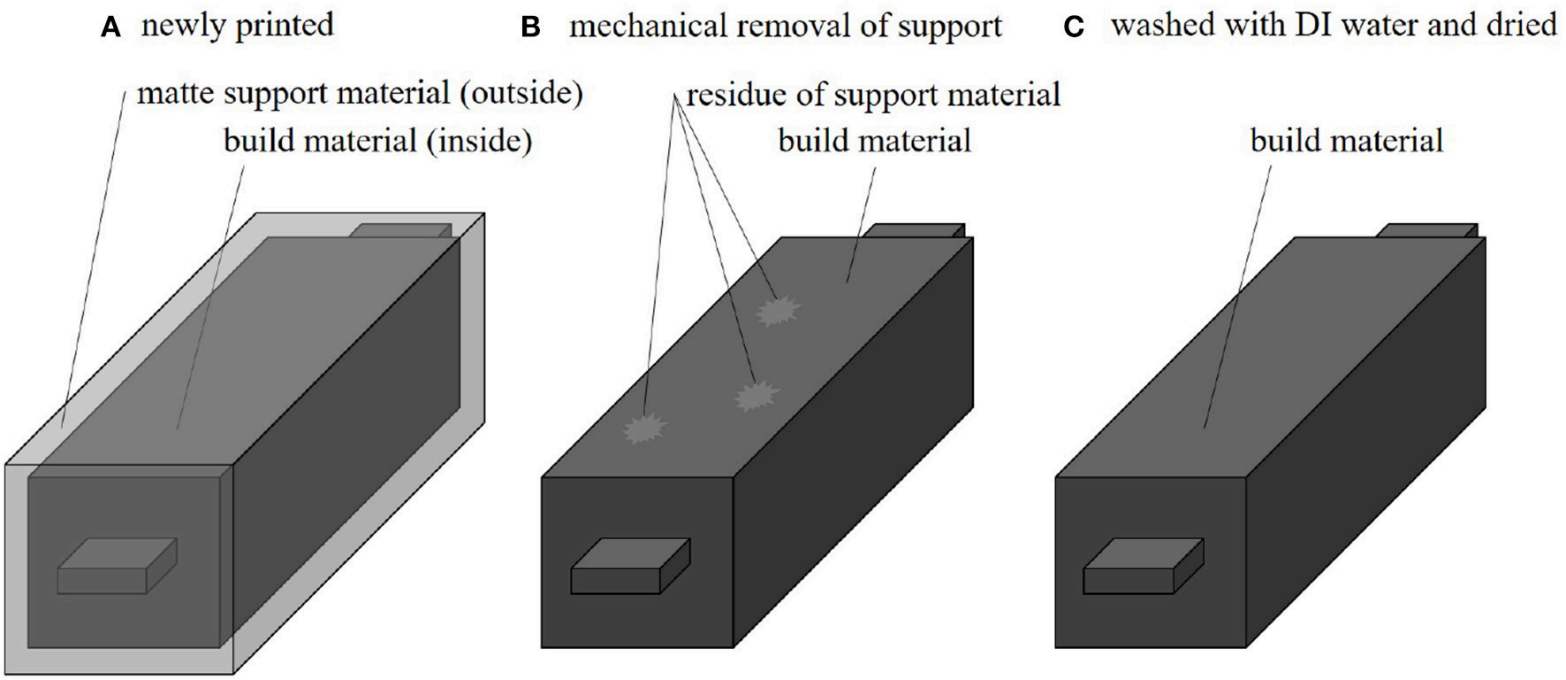
Stages of the process for removing the support material. **(A)** Newly printed block of material for characterization, with a matte surface finish. **(B)** Block of material after we mechanically remove the support material. Some patches of support material can remain on the surface. **(C)** Block of material after we wash it with distilled water and dry the sample.

Materials that the printer can produce include a flexible, translucent photopolymer (TangoPlus FLX930); a flexible, black photopolymer (TangoBlackPlus FLX980); a rigid, clear photopolymer (VeroClear RGD810); and a flexible, low-yield polymer (SUP705) as support material. The black resin contains carbon particles (Stratasys, 2014b), which provide a small but measurable conductivity, and can serve as conductive traces for sensors. We created the geometry of the sensors using computer-aided design (CAD) software, fabricated our designs using the 3D printer, and secured the wires using silver paste and mechanical strain reliefs (Figure 1). Figures 2, 3 show the sensor-only designs and the test samples, respectively, that we characterized in this paper.

The commercial material has comparable mechanical strain-to-failure properties as skin: it is reported to have 70–120% elongation at break (Stratasys, 2016). During our tests (section 3) we observed strain-to-failure of approximately 20%. However, even this reduced value may be sufficient for most applications. For comparison, most types of human skin fail after exceeding 15% strain (Kim et al., 2017). The physiological limitation is measured to be at most 45% in areas such as the fully flexed knee or elbow (Wessendorf and Newman, 2012; Kim et al., 2017). Thus, even with relatively low strain-to-failure, the commercial material may still be useful in creating bioinspired and biomimetic designs.

### 2.2. Removal of Support Material

The printer allows the user to choose between surface finishes that are either glossy or matte, where matte is achieved by coating the entire print in support material. We printed the test blocks with matte coatings to maintain a uniform surface finish. The dimensions of the test blocks were 10 × 10 × 50 mm, with 10 × 10 × 3 mm tabs for the measurement clamps (Figure 3). The process we use to remove the support material occurs in multiple stages (Figure 3). Because the support material appears to contribute to the conductivity (further evaluated in section 3.4), it is important to thoroughly clean the surface of the material or enclose it within a different material. In this process (Figure 3A), a fresh block of material comes off the 3D printer with a matte surface finish and is coated in support material. Figure 3B depicts the block of material after the support is mechanically scraped off the surface of the block by hand. Finally, as shown in Figure 3C, this process ends with the careful cleaning of any remaining support material on the surface of the block, by washing the block with distilled water and drying it.

### 2.3. Mechanical Strain Relief

As noted in previous work (Mengüç et al., 2014; Shih et al., 2018), attaching solid-core wires to soft materials can be difficult and result in an unstable connection because the wire can tear the soft material or gradually shift around. Thus, our solution for effective electrical connections is to combine: (1) soft insertion points for mechanically securing the wires, and (2) additional mechanical relief using an extra loop of wire to wrap around, which reduce the tearing of the electrodes when the sensor experiences strain.

**Figure 4:**
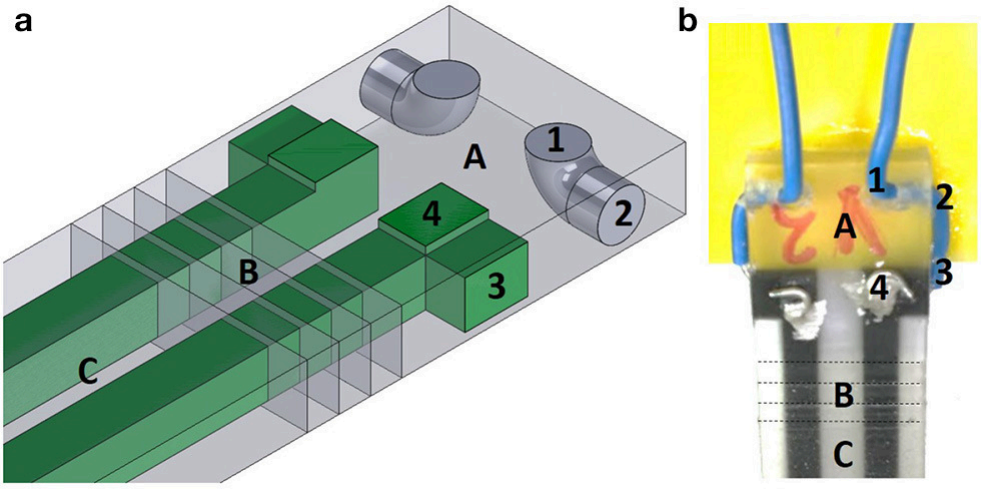
Our method for wire attachment. **(a)** Schematic for the wire attachment procedure - isometric view. We insert the wire at 1, and pass through in order of 1-2-3-4 to both distribute strain on the wire and to improve the electrical conductivity between the solid wire and the photopolymer trace. These numbers are only labeled on half of the sensor shown, for clarity. **(b)** Completed, physical version of the wire attachment - top view.

For this sensor design, we included soft holes within the rigid material to function as a mounting point. The hole in Figure 4 connecting the points labeled 1 and 2 is filled during the printing process using the dielectric photopolymer (TangoPlus, FLX930), which provides mechanical relief by restricting the motion of the wire. At 3 and 4, we push the bare wire through the black elastomer and coat the wire and photopolymer interface with silver paste to increase electrical conductivity. In Figure 4, A represents a rigid photopolymer (VeroClear RGD810, Stratasys), B represents a functional stiffness gradient (consisting of TangoPlus and VeroClear: FLX9050, FLX9070, and FLX9095, Stratasys), and C represents a flexible photopolymer (TangoPlus FLX930, Stratasys).

### 2.4. Experimental Setup for Measurement and Characterization

We connected the sensors to an inductance, capacitance, and resistance (LCR) meter (Keysight, E4980AL), which provides high precision measurements. We smoothed the sensor readings with an 8 point averaging filter on the LCR meter.

**Figure 5:**
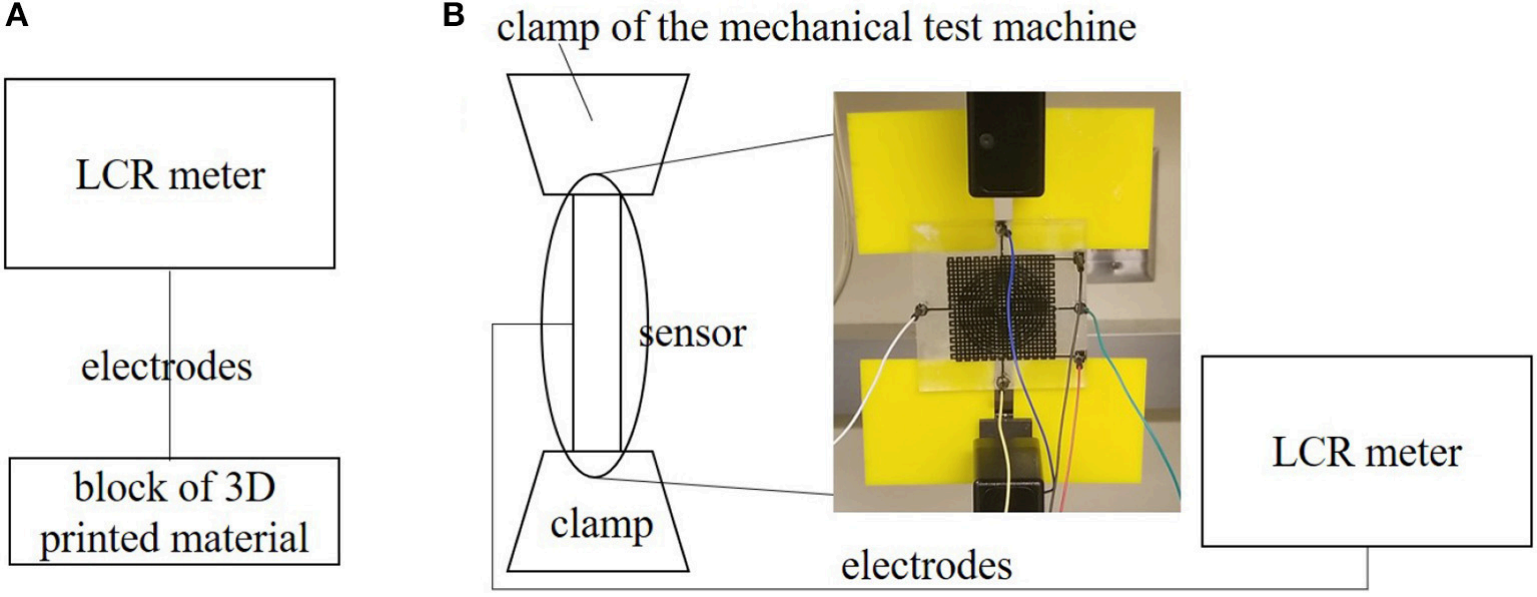
Test setup for characterization. **(A)** Setup to measure conductivity of blocks. **(B)** Setup to simultaneously measure strain and conductivity.

We prescribed the strain using a mechanical testing machine (Instron 5965) (Figures 5A, **10**, **11A**). For the multilayer sensor, we tested the pressure by placing a range of standard masses on top of the pressure sensor, which we converted to a pressure using the relationship ($P=\frac{mg}{A}$) (Figure 11B).

### 2.5. Removal of Water Content by Desiccation

Because of the constituent components in the 3D printer material (further explanation in section 3.4), we investigated how much of an impact water content had on mass and conductivity. In the desiccation process, we placed the test blocks in an air-tight, sealed container with packets of silica desiccant (Dry & Dry). Throughout multiple 1 h periods, we removed the blocks and reported the conductivity.

### 2.6. Preferential Strain Response of the Multi-Dimensional Sensor

We looked at the sensitivity of the multi-directional sensor by connecting it to the LCR meter while simultaneously stretching the sensor in the Instron. The parameters of the Instron include a strain rate of 0.0025 mm/mm/s (0.25% strain per second) and an auto-stop at 15% extension. Figure 5B shows the setup for simultaneously stretching the material while measuring the conductivity.

### 2.7. Model of Strain Response

For elastomer-like materials, the overall length of the channel increases while the cross-sectional area of the channel decreases when the channel of the sensor experiences axial deformation, leading to an increase in the resistance of the channel (Park et al., 2012). Here, we are ignoring the microscale, bond-level effects (Creton and Ciccotti, 2016) and assuming the change is dominated by macroscopic geometric change.

Assuming rectangular traces, we can represent the resistance of an undeformed sensor as:

(1) $$\begin{array}{r} \begin{array}{c} R_{0}=\frac{\rho L}{wh} \end{array} \end{array}$$

where *R*_0_ is the resistance in the undeformed state, ρ is the electrical resistivity of the photopolymer, L is the length of the conductive channel, and *w* and *h* are the width and height of the cross-section of the conductive material, respectively.

The change in resistance of the stretched material is:

(2) $$\begin{array}{lll} \Delta R & = & R-R_{0}\\ & = & \rho \frac{L+\Delta L}{\left(w+\Delta w)(h+\Delta h\right)}-\rho \frac{L}{wh} \end{array}$$

where *R* is the resistance when the sensor stretches by Δ*L*.

Next, we can replace Δ*w* with −ν*ϵw* and Δ*h* with −ν*ϵh*, and substitute $\epsilon =\frac{\Delta L}{L}$ to obtain:

(3) $$\begin{array}{r} \begin{array}{c} \frac{\Delta R}{R_{0}}=\epsilon \left[\frac{\left(1+2\nu \right)-\nu^{2}\epsilon }{{\left(1-\nu \epsilon \right)}^{2}}\right] \end{array} \end{array}$$

where ϵ is the strain and ν is the Poisson's ratio.

The Poisson's ratio for this photopolymer material is approximately ν = 0.49 (Slesarenko and Rudykh, 2017), which enables us to further simplify Equation 3 to:

(4) $$\begin{array}{r} \begin{array}{c} \frac{\Delta R}{R_{0}}=\frac{\epsilon \left(8.25-\epsilon \right)}{{\left(2.04-\epsilon \right)}^{2}} \end{array} \end{array}$$

### 2.8. Model of Pressure Response

We modeled the relationship between the change in resistance and contact pressure using linear elastic fracture mechanics (Park et al., 2010). Once again, assuming rectangular traces, we can represent the normalized change in resistance as:

(5) $$\begin{array}{r} \begin{array}{c} \frac{\Delta R}{R_{0}}=\frac{1}{1-2\left(1-\nu^{2}\right)wP/\left(Eh\right)}-1 \end{array} \end{array}$$

where *P* is the pressure on the sensor and *E* is the elastic modulus of the material.

## 3. Results and Discussion

### 3.1. Characterization of the Dependence of Conductivity on Orientation of 3D Print

Previous work has shown that 3D printers exhibit anisotropic properties depending on the orientation of the print (Wang et al., 2017a). Thus, we characterized the dependence of the conductivity on the orientation of 3D print. We tested samples of each of the TangoPlus, TangoBlackPlus, and VeroClear materials.

**Figure 6:**
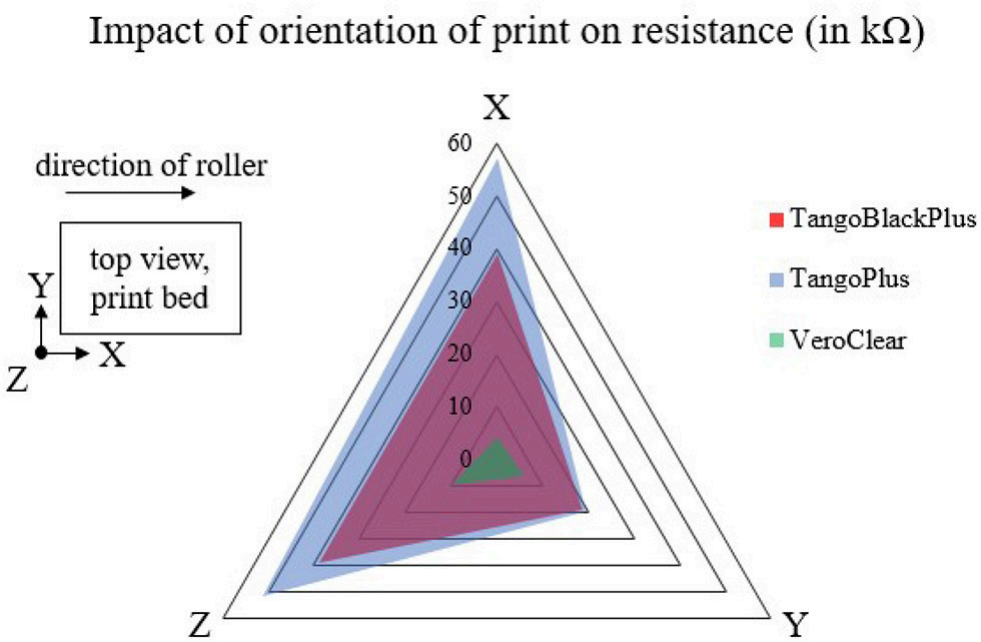
Plot of the impact of anisotropy on the 3D printed material. Number of samples *n* = 10.

Our results (Figure 6) show that there is a difference in resistance measurements between TangoBlackPlus and TangoPlus in the x-axis and z-axis directions of the 3D print. However, in the y-axis direction, the conductivity changes minimally between the two materials. In all three directions of the coordinate frame, there is a substantial difference between the readings for the VeroClear and for the other materials.

This phenomenon may be due to the spacing of the print heads. The commercial 3D printer moves along the x-axis of the bed and sputters droplets of uncured ink onto the print bed. The printer cures each layer of ink with a UV light before incrementing the positioning of the z-axis and moving to the subsequent layer. In addition, the roller component of the 3D printer, which smooths each layer of ink, also acts along the x-axis. This asymmetry in the plot could be due to increased overlapping of the ink in the x direction relative to the y direction. For the remainder of the experiments, we consider only the conductivity of the in-plane x-direction.

### 3.2. Characterization of the Dependence of Conductivity on Removal of Support Material

To characterize the dependence of conductivity on the amount of support material coating the surface of the blocks of material, we measured the resistance of the materials at various points throughout the process of removing the support material.

**Figure 7:**
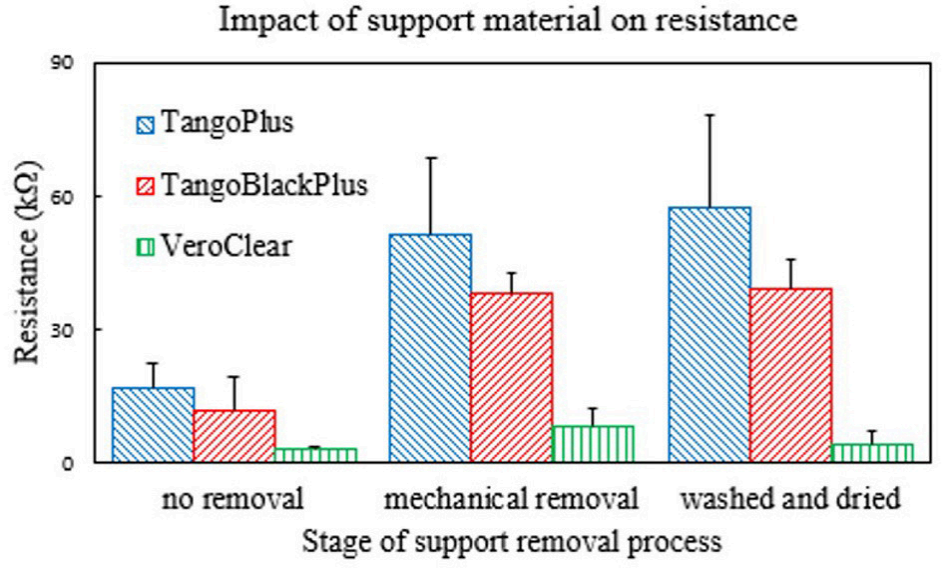
Plot of how the resistance of the 3D printed materials changes depending on how much support material is on the surface. Each group of bars corresponds to the measurement along the various stages in the removal process of the support material (Figure 3). Number of samples *n* = 10.

We observed that the resistance of the TangoBlackPlus and TangoPlus samples increased, meaning the conductivity of the samples decreased overall (Figure 7). This observation suggests that the support material contributed toward the conductivity measurement of each material, and indicated that the surface of various materials need to be thoroughly cleaned of support material.

In addition, the data show that the VeroClear material actually exhibited the most conductivity. While this result is interesting, we cannot directly integrate it into soft sensors or soft-bodied robots because of its intrinsic rigid mechanical properties. However, the relatively-lower resistance of VeroClear may be useful for other applications.

### 3.3. Characterization of the Dependence of Conductivity on Contact Resistance

**Figure 8:**
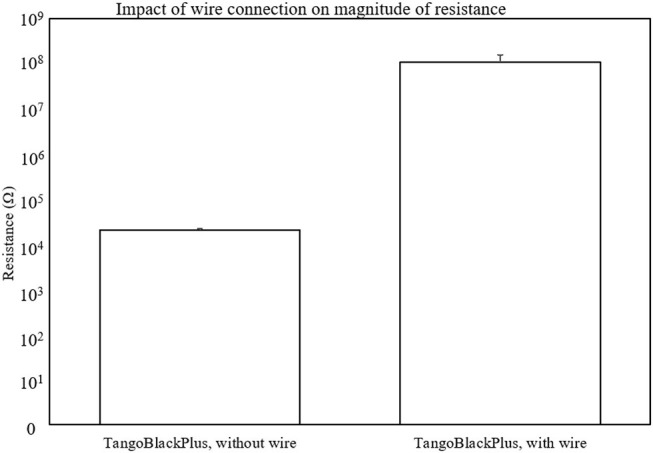
Plot of the dependence of the magnitude of conductivity based on contact resistance. We plot the magnitudes on a logarithmic scale to show the order of magnitude difference that the wire connection results in. Number of samples *n* = 3.

While measuring the materials and designing the mechanical strain relief, we observed that the contact resistance due to the rigid wire and soft material appeared to play a significant role in the measurement of the conductivity. Thus, we characterized the dependence of conductivity on the contact resistance by comparing the material with and without wires (clamping onto the material directly with the LCR probes). Figure 8 shows the results of the comparison of the resistance pre- and post- wire attachment. Because the system is soft, it deforms upon clamping. We avoided directly clamping to the soft material by attaching a wire. The resistance measured when clamping directly to the material is less than that measured when a wire is connected to the material with silver paste at the interface because of contact resistance that the wiring introduced.

When the material comes directly into contact with the clamps for measurement, it gradually tears due to a stress concentration at the interface of contact. The mechanical breakdown of the material reinforces the need for a soft-rigid wire interface. By adding wires to the samples, we could reduce variance in the LCR measurements by providing a fixed point of attachment to the soft material. A single, physical point of contact also helped with the consistency of the measurements by reducing the mechanical wear of the LCR electrodes on the samples. Additionally, the mechanically strain relief (section 2.3) further helps with reducing the movement of the wires.

There may be two scales of physical behavior that influence the reading that the LCR meter produces: (1) the physical, geometric deformation of the material at the macro-scale, and (2) the micro-scale separation of the particles in the soft material as the chemical bonds between the particles begin to separate and the material begins to tear (Creton and Ciccotti, 2016).

### 3.4. Characterization of the Dependence of Conductivity on Water Content

The datasheets for the 3D printed materials indicate that they contain hydrophilic, organic compounds (Stratasys, 2014a,b,c,d). In addition, we noticed that the cleanliness of the surface of the materials, particularly the removal of the support material, had an impact on the measurement of conductivity. These observations indicated that the support material was contributing to the conductivity, possibly because of the ability of the material to absorb and hold onto water.

**Figure 9:**
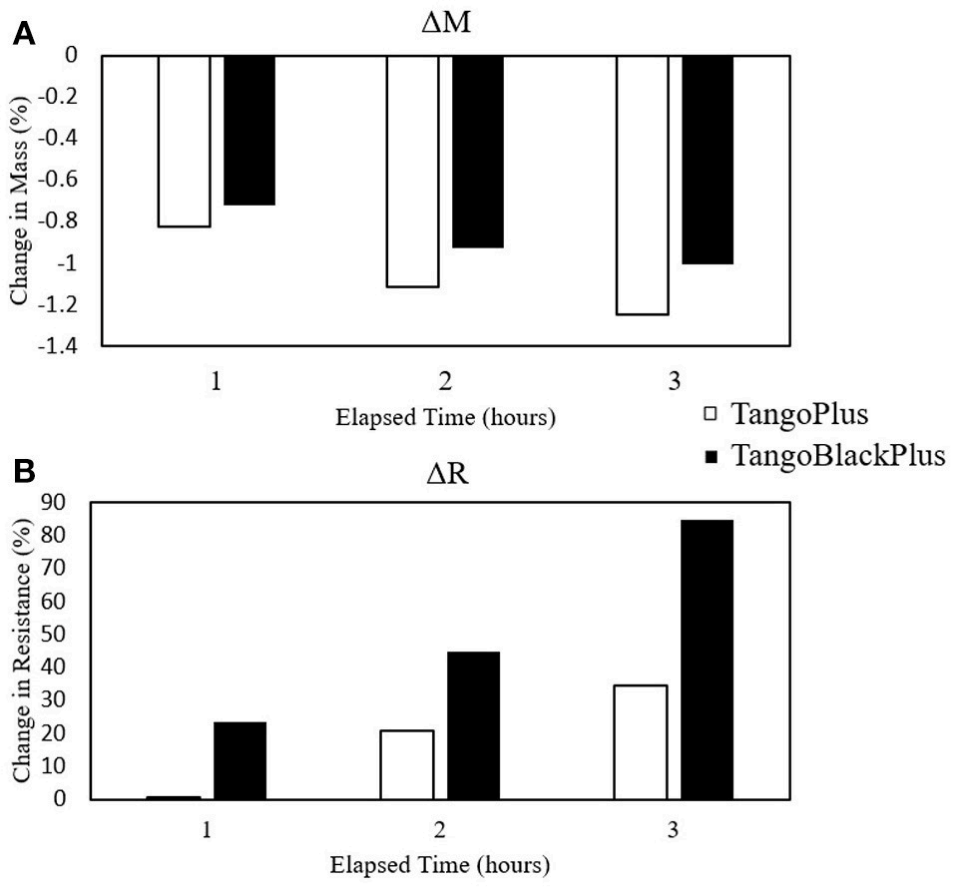
Plots of changes in mass and resistance due to desiccation. **(A)** Percent change in mass. **(B)** Percent change in resistance. Number of samples *n* = 6.

To further understand how the water content impacts the conductivity of the material, we tested the effects of desiccating the materials. We reduced the water content in the blocks using silica desiccants, and measured the changes in mass due to water content as well as the resulting change in conductivity (Figure 9). This procedure is further described in section 2.5.

The mass of the blocks decreased due to the desiccation, which we attribute to the loss of water content. The desiccation process resulted in an overall decrease of –1.00%. The average resistance of the samples from the desiccation process increased by 84.78%, indicating that the samples became less conductive than before the desiccation process and that the presence of moisture can impact the sensor readings.

### 3.5. Comparison Between Model and Experimental Data

**Figure 10:**
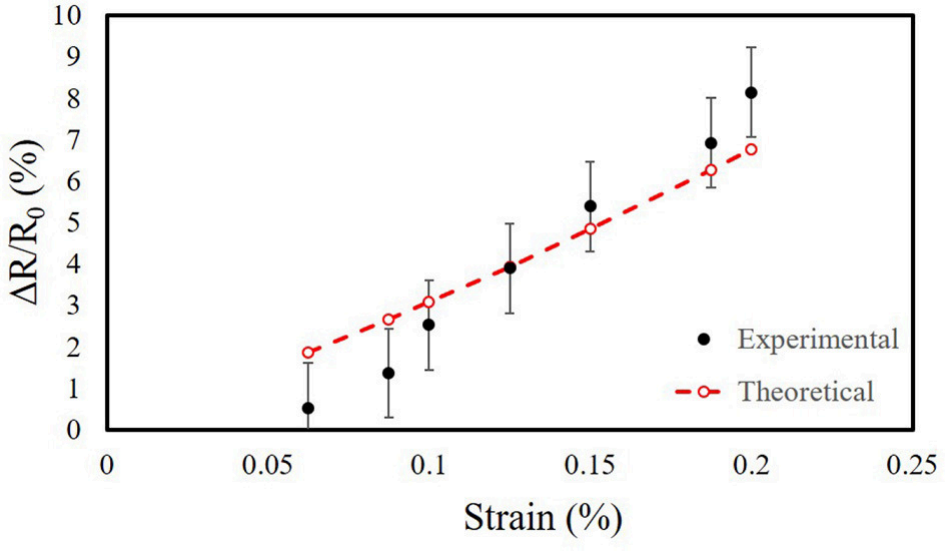
Normalized change in resistance vs. strain for the uniaxial strain sensor. Comparison of analytical model to experimental characterization. The mean is 108 MΩ at rest. Number of samples *n* = 3.

**Figure 11:**
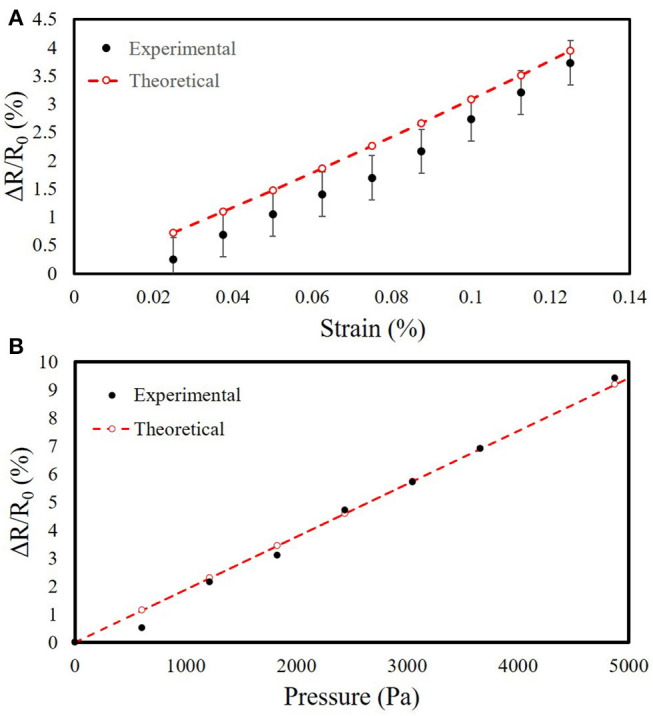
Comparison of analytical model to experimental characterization for the multilayer sensor. **(A)** Normalized change in resistance vs. strain. The mean is 147 MΩ at rest. Number of samples *n* = 3. **(B)** Normalized change in resistance vs. pressure. The mean is 560 MΩ at rest.

We compared the analytical models of the uniaxial and multilayer sensors (section 2.7) with the measurements from our experimental tests. We characterized our sensors using an LCR meter. We required sensitive measurement equipment because the conductive photopolymer has a carbon black percentage of < 0.1% by mass (Stratasys, 2014b), resulting in resistances with large magnitudes (in the MΩ range). We filtered the sensor readings with a 32 point averaging window on the LCR meter. Figure 10 shows the normalized change in resistance vs. strain for the uniaxial strain sensor, Figure 11A shows the normalized change in resistance vs. strain along one of the two axes of strain for the multilayer sensor, and Figure 11B shows the normalized change in resistance vs. pressure for the multilayer sensor.

To better visualize the trends, we introduced a single constant scale factor of *S*_*strain*_ = 14.25 for both strain sensors (Equation 6) and another of *S*_*pressure*_ = 3, 850 for the pressure sensor (Equation 7), resulting in the theoretical plots in Figures 10, 11. For the scale factor for strain, we selected its value by simultaneously minimizing the residuals for both the uniaxial and multilayer sensors. The scale factors are included to demonstrate that despite the differences in magnitude, the trend of the measurements behaves similarly to predictions of the sensors with similar geometry of the traces despite differences in the materials for fabricating the sensors.

(6) $$\begin{array}{r} \begin{array}{c} \frac{\Delta R}{R_{0}}=S_{strain}\epsilon \frac{\left(8.25-\epsilon \right)}{{\left(2.04-\epsilon \right)}^{2}}\\ \frac{\Delta R}{R_{0}}=S_{pressure}\left[\frac{1}{1-2\left(1-\nu^{2}\right)wP/\left(Eh\right)}-1\right] \end{array} \end{array}$$

One potential reason for the difference in scale is that the resistivity may not be constant as we had previously assumed. During stretching, the geometry of the sensor changes, which can be described with Poisson's ratio. This change in geometry alters the resulting resistance of the trace. However, since our traces are polymers, strain will cause some carbon particles to get closer together while others will separate, depending on the initial distribution of the polymer network within the dielectric (Zhang et al., 2007). This would impact electron mobility, and thus the assumption of constant resistivity might not be valid and requires further investigation.

### 3.6. Multimodal Sensitivity

Analysis of the strain response of the multi-layer sensor helped identify that the patterning of the traces make a difference in the measurement, and demonstrated that the LCR meter could discern between the different directions of strain.

**Figure 12:**
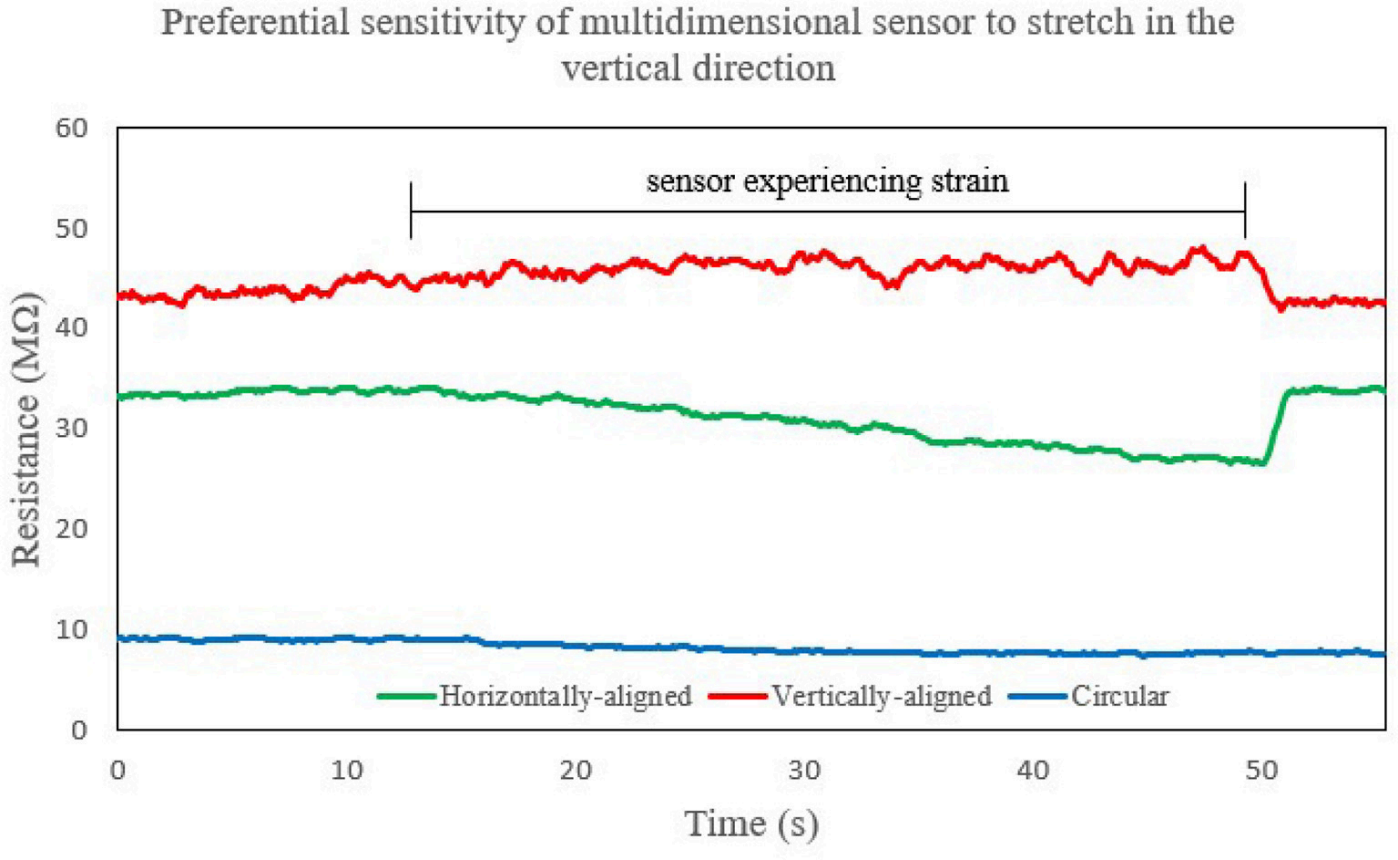
Plot showing the strain response of the multimodal sensor in multiple directions as we stretch it along the vertically-aligned direction. In this situation, the sensor is more sensitive to the serpentine pattern that is horizontally-aligned as opposed to the one that is vertically-aligned, whereas the circular sensor experiences a minimal change in its response relative to the other directions.

When stretching in the vertically-aligned direction, we observed that the vertically-aligned sensor increased in resistance whereas the horizontally-aligned and pressure sensors decreased in resistance (Figure 12). The trend that we observed is consistent with what we expected from Poisson's ratio. However, the magnitudes are not: the vertically-aligned traces experienced less relative change than the horizontally-aligned traces did. This effect could potentially be because the change in resistance is bigger when you compress the material together than when you stretch it apart, as compared to liquid materials such as eGaIn. Modeling this material as a conductor of changing volume is not accurate, but rather the material is more sensitive to compression than extension as shown from these results.

### 3.7. 3D Printed Soft Gripper With Embedded Sensors

**Figure 13:**
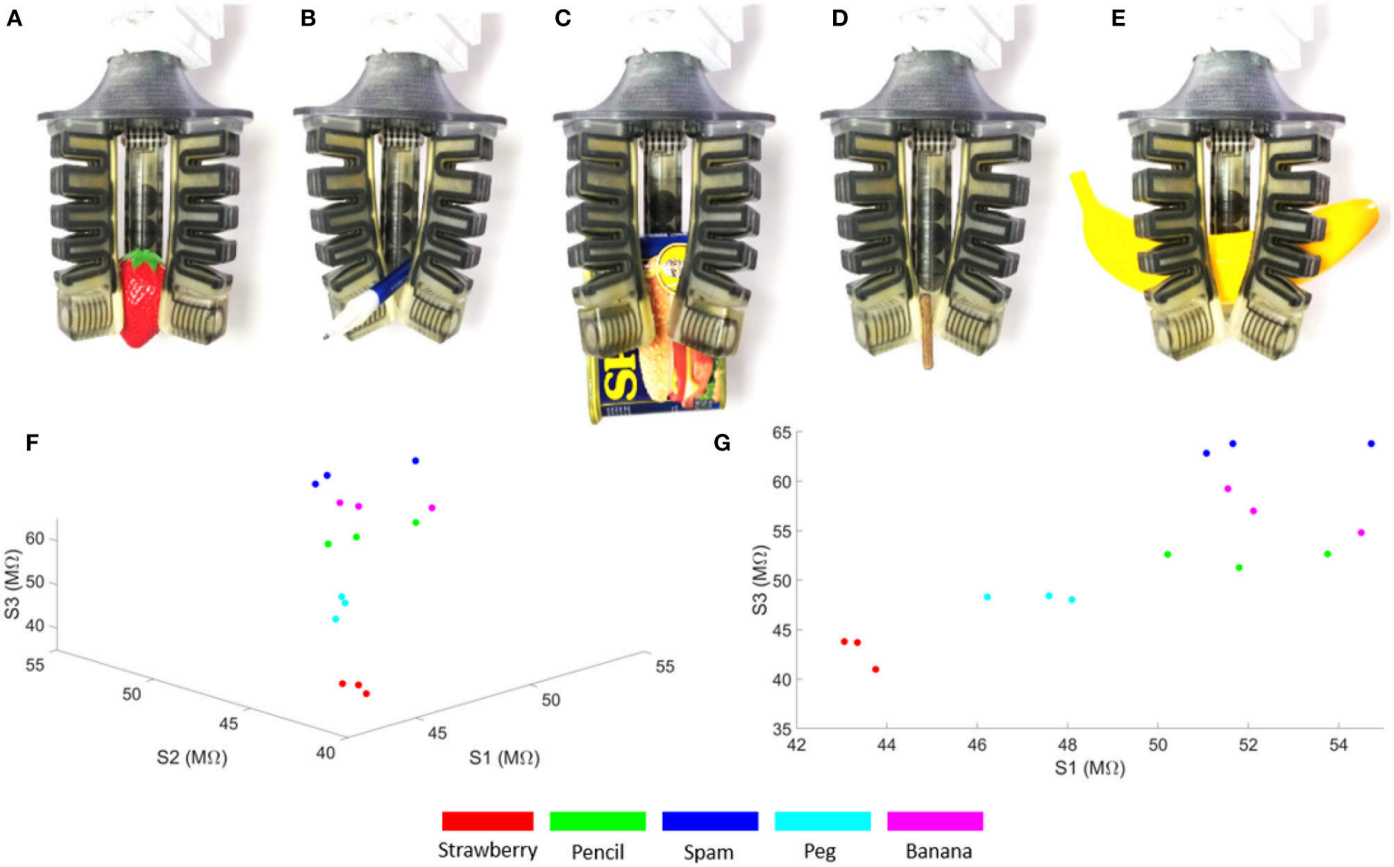
Pneumatic gripper with embedded strain sensors and the static sensor readings of the gripper corresponding to the configuration of the fingers while holding various objects. S1, S2, and S3 are the sensor readings from each of the three fingers. **(A)** Plastic strawberry. **(B)** Pencil. **(C)** Can of spam. **(D)** Toy peg. **(E)** Plastic banana. **(F)** 3D perspective of the scatter plot. **(G)** 2D perspective of the scatter plot.

**Figure 14:**
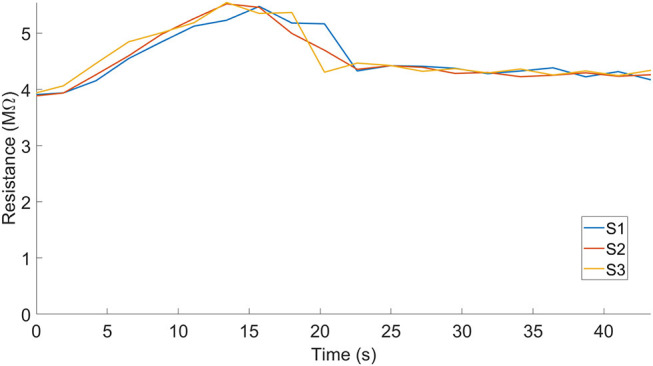
Dynamic sensor readings of the gripper as the fingers close, without an object. S1, S2, and S3 represent each of the sensor readings that the fingers in the gripper produced.

We demonstrated the use of this fabrication process to print a soft gripper with embedded sensors by integrating the sensing capabilities directly into a pneumatic gripper (Figure 13). In addition, we measured the static sensor readings as the gripper held onto various objects, which we selected from the YCB dataset (Calli et al., 2017) (Figure 13). We also measured the dynamic response of the sensors as we actuated the fingers simultaneously (Figure 14).

As a proof of concept demonstration, we measured the sensor readings for the various gripper configurations associated with grasping different objects. Each finger is pneumatically actuated, like previously presented pneunets (Mosadegh et al., 2014), and we denoted the corresponding sensors as S1, S2, and S3. We defined holding as the grasp position associated with the configuration of each finger as it conforms around the objects (Figure 13) and repeated each grasp 3 times. Previous work has shown that these configurations can be used to identify or classify these object-associated grasps (Homberg et al., 2015). The embedded sensors could be beneficial for detecting touch or helping with state estimation for the gripper, and clusters formed from multiple grasps could be amenable to classification.

## 4. Conclusions and Future Work

This paper presents the concept of 3D printing resistive sensors using a commercially available printer and material. We characterized the dependence of the 3D printed material's conductivity on a variety of factors and introduce guidelines on how to improve the fabrication method for similar types of sensors. In addition, we studied the mechanical strain response and model the sensors that we fabricated using this process.

The current iteration of the 3D printed material is promising, but there are some limitations to consider when working with it. To begin with, the company did not intentionally design the material as a conductor, and thus the resistance has a large magnitude. Measurement of the sensor values requires high-sensitivity electronics and the high impedance and limited conductivity could limit sensitivity. The readings also experience drift and oscillations due to the macro- and micro-scale deformations of the material. In addition, this photopolymer material experiences limited strain compared to materials for “traditional” soft sensors like silicone elastomers, which could limit its use for soft robots.

Future work includes studying the other modes of sensors that this method may enable, such as printing capacitive and inductive sensors. Finding a method to reduce the drift of the readings over time would help improve the potential for deploying this material for real-world applications. In addition, knowledge of the mechanical and chemical properties of the materials can inform and optimize the design of 3D printed sensors for future applications. The ability to embed sensors into soft robots using accessible, commercial 3D printers would 1 day play a role in printing an entire robot along with all of its components.

This work is a step toward the direct printing of sensorized soft robots using commercially available systems. Simultaneously, embedded printing of sensors is a powerful process that could enable and enhance seamless integration of sensors into soft robots, but there does not yet exist a suitable, commercially available, easy-to-use platform that allows users to simultaneously print soft actuators and sensors. Because we expect the availability of conductive, commercial 3D printed materials to increase in the future, our focus for the future can shift to further exploring applications that this approach enables. As 3D printing technology improves and decreases in cost, multimaterial and conductive printing will grow increasingly ubiquitous and accessible, further enabling novel methods for integrating sensors into complex bodies.

Multimaterial digital manufacturing has the potential to enable robot designs with not only varying mechanical properties, but also varying electrical properties. This capability enables a whole new design space of sensors embedded in the bodies of soft robots. However, there are currently several practical challenges to applying this method, including interfacing and characterization of material properties. In this paper, we explored some of the above challenges and demonstrated how we can fabricate soft, fully 3D printable machines with embedded sensing.

## Author Contributions

BS conceptualized the project, designed the experiments, performed characterizations, analyzed the model, interpreted the results, and wrote the paper. CC conceptualized the project, performed characterizations, designed the experiments, and interpreted the results. KG, SL, JM, and ZH refined the fabrication process and helped with experiments. MT oversaw all activities.

### Conflict of Interest Statement

The authors declare that the research was conducted in the absence of any commercial or financial relationships that could be construed as a potential conflict of interest.
